# Contraceptive Care Visit Objectives and Outcomes: Evidence From Burkina Faso, Pakistan, and Tanzania

**DOI:** 10.1111/sifp.12279

**Published:** 2024-12-03

**Authors:** Corrina Moucheraud, Alexandra Wollum, Mohamad Brooks, Manisha Shah, Jessica Gipson, Zachary Wagner

**Affiliations:** ^1^ New York University School of Global Public Health, New York, NY 10003, USA; ^2^ University of California Los Angeles Fielding School of Public Health, Los Angeles, CA 90095, USA; ^3^ Pathfinder International, Watertown, MA 02472, USA; ^4^ University of California Berkeley Goldman School of Public Policy, Berkeley, CA 94720, USA; ^5^ Center for Economic and Social Research, University of Southern California, Los Angeles, CA 90089, USA

## Abstract

Globally, care experiences of the growing population of contraceptive users are not well‐understood. We leverage a large client dataset (*n* = 71,602) from three countries (Burkina Faso, Pakistan, and Tanzania) to characterize contraceptive services sought (visit objective and method preference), assess whether these visit objectives were met and for whom, and explore if visit objective fulfillment was associated with care quality. Most people in all three countries said they were seeking to continue their current method or adopt a method for the first time. Clients seeking to change their method were least likely to have their objective met: 63.7 percent of clients in Burkina Faso, 73.3 percent in Pakistan, and 61.1 percent in Tanzania who wanted to switch actually achieved this during the visit. In Burkina Faso, people with lower socioeconomic standing, lower educational attainment, and lower parity less commonly had their switching objective, fulfilled. Method preference fulfillment was generally high, although approximately 15 percent of Tanzanian clients were given implants despite wanting another method. Among those seeking to adopt or restart a method in Pakistan and Tanzania, having this visit objective fulfilled, was correlated with better perceived treatment and higher person‐centeredness of care.

## BACKGROUND

Nearly a billion women worldwide use modern methods of contraception, an increase of 42 percent since 1990 (United Nations Department of Economic and Social Affairs 2020). As the use of modern methods increases, so too will the number, who want to switch or discontinue methods (Howett et al. 2019; Bertrand et al. 2020; Sergison et al. 2017). People who are using contraception for birth spacing will want to discontinue when they wish to resume childbearing. Additionally, those who experience side effects, or whose preferences change as their fertility intentions evolve over the life course (Trinitapoli and Yeatman 2018; Sennott and Yeatman 2012; Yeatman, Sennott, and Culpepper 2013), may want to change methods to better meet their needs.

For many people, this decision will require interaction with the health system: someone using a long‐acting reversible contraceptive (LARC), like an implant or an intrauterine device (IUD), often needs assistance from a healthcare worker when they want to switch their method or discontinue use, as do people seeking to switch to or between modern methods including LARCs, oral contraceptive pills, and injectable contraceptives (Bertrand et al. 2020).

However, the global health community is ill‐prepared for this increased demand for switching and discontinuation services (Christofield and Lacoste 2016; Ali, Folz, and Farron 2019; Bryson, Koyama, and Hassan 2021; Wollum, Moucheraud, Sabasaba, et al. 2024). Many initiatives—and corresponding donor funds and monitoring targets—focus on increasing the uptake of family planning, but few attend to the unique needs of those who want to switch or discontinue their method. The limited available evidence points to challenges in ensuring high‐quality care for people seeking switching or discontinuation (Callahan et al. 2020; Britton et al. 2021; Senderowicz et al. 2022). This is a major gap in our understanding of how to deliver person‐centered, effective, equitable family planning services (Jacinto et al. 2022; Starrs et al. 2018).

In this paper, we leverage a unique dataset of exit surveys conducted with family planning clients in Burkina Faso, Pakistan, and Tanzania (Pathfinder International 2019) (*n* = 71,602) to characterize the visit objectives of family planning clients, to estimate what share of clients have their visit objectives met, and to assess whether quality of care differs by whether objectives are met or not. This concept of “visit objective fulfillment”—that is, whether a client's stated desired objective for a family planning service visit matches the outcome of that visit—emphasizes a client's preferences when considering contraceptive service provision. We see this concept as related to other measures of contraceptive preference and autonomy in that it takes a cross‐sectional, person‐centered approach to measure whether someone's contraceptive preferences are met (Holt et al. 2023; Senderowicz 2020; Sokol et al. 2024; Burke and Potter 2023); the analysis presented here focuses on the contraceptive service point at a health facility, so is somewhat more narrowly focused on the care encounter and its role in the translation of preferences to use or nonuse. This measure adds to the growing body of literature that aims to ask people about their family planning preferences instead of assuming this preference based on fertility intentions (e.g., as is assumed in measures of unmet need) (Speizer, Bremner, and Farid 2022; Fabic 2022; Bhan and Raj 2021; Burke and Potter 2023). We also explore visit objective fulfillment by the contraceptive method used by the client, and the contraceptive method preferred by the client—and these allow a more nuanced exploration of groups seeking to switch or discontinue contraceptive methods, and of potential method biases. Of note, the concept of “visit objective fulfillment” is value‐neutral, that is, it is agnostic to whether visit objective fulfillment is a positive outcome; there are myriad reasons why a client's visit objective may be unmet, including that their preferences for any method, or a specific method, may update during the visit as they receive additional information.

## METHODS

### Parent Study

The Beyond Bias project was a cluster‐randomized controlled trial to address sources of family planning provider bias, implemented by Pathfinder International in Burkina Faso, Pakistan, and Tanzania. Data from the Beyond Bias project to date have primarily been used to evaluate the intervention (Wagner et al. 2023a); there are also published reports about the design of the intervention (Murithi et al. 2021; Camber Collective. 2022; Murithi, Gibbs, and Hope 2022). These data provide a unique opportunity to explore additional research questions about contraceptive service provision, such as this one.

This analysis uses data collected during the Beyond Bias project between September 2020 and August 2021, through exit surveys with family planning clients at 227 health facilities across three countries (Wagner et al. 2023a): 73 public health facilities in Tanzania (in its largest city, Dar es Salaam), 76 private health facilities in Pakistan (in its largest city, Karachi), and 78 public health facilities in Burkina Faso (in urban and peri‐urban areas around Ouagadougou, Banfora, and Bobo). These facilities were selected because they had an existing relationship with Pathfinder International, the implementing organization of the Beyond Bias intervention, and as such should not be treated as a representative sample of facilities in the respective geographies.

The exit surveys were conducted with female family planning clients, both new and existing users; youth enumerators aimed to approach all clients after they had completed their visit with a provider to participate in the survey. Enumerators read survey questions aloud and input responses on a tablet. Surveys lasted between 10–15 minutes. All countries implemented the same survey instrument (with some small adjustments for language and cultural considerations). The sample for this analysis included all exit surveys with people who said they had visited the facility for family planning services (excluding those who received family planning services but had not visited the facility for that purpose), and for whom we could define a visit objective or visit outcome. For clients who were using a method at the time of their visit, we limit our analysis to those who came to the facility that day using, or left the facility using, an implant, an IUD, an oral contraceptive pill, or a contraceptive injection; 95 percent of our sample met this requirement.

### Study Setting

The study countries are described in Table 1. They range in population size, from approximately 5 million people of reproductive age in Burkina Faso to over 57 million in Pakistan. On average, a woman in Pakistan bears 3.47 children, which is lower than the total fertility rate in Burkina Faso and Tanzania (4.77 and 4.73, respectively); women in Pakistan have a later sexual debut (age 20.7 on median) than in Burkina Faso (age 17.7) or Tanzania (17.2). In Pakistan, 28.1 percent of women aged 15–49 report using a modern method of contraception, as do 30.4 percent in Burkina Faso and 38.5 percent in Tanzania.

**TABLE 1:** Characteristics of included countries

	Burkina Faso	Pakistan	Tanzania
Global region and income group (World Bank 2020)	Africa; Low‐income economy	South Asia; Lower‐middle income economy	Africa; Lower‐middle income economy
Female population aged 15–49 years, 2021 (United Nations 2022)	5.20 million	57.25 million	15.31 million
GDP per capita (PPP, constant 2017 I$), 2021 (World Bank 2017)	$2,180	$5,232	$2,582
Total fertility rate, 2021 (United Nations 2022)	4.77 births per woman	3.47 births per woman	4.73 births per woman
Adolescent fertility rate, 2021 (United Nations 2022)	110.5 births per 1000 women ages 15–19	42.3 births per 1000 women ages 15–19	123.7 births per 1000 women ages 15–19
Contraceptive prevalence among women aged 15–49, any modern method, 2021 (United Nations 2022)	30.4%	28.1%	38.5%
Current use of pill^a^ (Institut National de la Statistique et de la Démographie ‐ INSD/Burkina Faso and ICF International 2012; National Institute of Population Studies ‐ NIPS/Pakistan and ICF 2019; Ministry of Health et al. 2016)	2.5%	1.7%	2.1%
Current use of IUD^a^	1.5%	2.1%	0.6%
Current use of injections^a^	6.5%	2.5%	7.0%
Current use of implants^a^	12.9%	0.4%	11.1%
Current use of male condom^a^	3.6%	9.2%	1.9%
Current use of female sterilization^a^	0.1%	8.8%	2.2%
Median age at first sexual intercourse (women aged 25–49), data year as noted	17.7 (2010) (Institut National de la Statistique et de la Démographie ‐ INSD/Burkina Faso and ICF International 2012)	20.7 (2017–2018) (National Institute of Population Studies ‐ NIPS/Pakistan and ICF 2019)	17.2 (2015–2016) (Ministry of Health et al. 2016)

^a^Measured among all women in Burkina Faso and Tanzania, and among married women in Pakistan.

### Typology of Family Planning Visits

We assessed the visit objective using the responses to questions that asked clients if they were using a contraceptive method prior to coming to the clinic, which contraceptive method they were using, whether they were visiting the clinic to continue/restart the same method (only current or previous [i.e., during their lifetime but not at the time of this visit/survey] users), whether they were seeking to get their method removed (IUD/implant users only), and whether they had a method in mind prior to their visit (for nonusers and clients who did not want to continue/restart using the same method they already used). Visit outcomes were measured using a question that asked clients whether and what type of method they received during their visit and whether their IUD/implant was removed during the visit (among those who said they were visiting the facility for a removal).

We define all family planning visitors based on the objective of their visit and the outcome of their visit (Table 2). We categorized clients into five different visit objective types: adopt, restart, continue, switch, or discontinue. For each visit objective type, we identified whether the objective was fulfilled based on the criteria in Table 2.

**TABLE 2:** Typology of family planning visit objectives (left) and visit outcomes (right)

Visit objective	Visit outcomes	
Objective type	Objective met	Objective not met
Intend to adopt: never used an eligible contraceptive method	Adoption: left visit with an eligible method	Nonadoption: did not leave visit with an eligible method
Intend to restart: not using an eligible contraceptive method currently but have used one previously	Restart: left visit with an eligible method	Nonrestart: left visit without an eligible method
Intend to continue using an eligible contraceptive method and want to continue the same method	Continuation: left visit with the same method	Switching: left visit with a different eligible method Discontinuation: left visit without a method
Intend to switch using an eligible contraceptive method did not want to continue the same, and wanted a different method	Switching: left visit with a different eligible method	Discontinuation: left visit without a method Continuation: left visit with the same method
Intend to discontinue using an implant or IUD, wanted it removed, and did not want another method	Discontinuation: reported their method was removed and left visit without a method	Switching: left with a different eligible method Continuation: left with the same method

### Outcome Variables

First, we explored visit objective fulfillment: Did the visit outcome match the client's stated visit objective? We looked at this by type of visit objective. We also investigated method preference fulfillment—i.e., did clients get the method they said they wanted at the start of the visit—within each objective type? We assessed method preference fulfillment using questions in the client exit survey that asked clients whether and which methods they had in mind before coming to the clinic and talking to a provider. For clients who wanted to continue using the same method, we assumed they preferred using their current method. We included clients who reported more than one preferred method in an “Other/multiple” category. This approach to assessing visit objective fulfillment and method preference fulfillment builds on previous work, such as Bullington et al.’s measure of nonpreferred contraceptive method use that compares a person's method use to their original preference (Bullington et al. 2023).

Second, we evaluated care quality by visit objective fulfillment. Care quality is a multidimensional concept (National Academies of Sciences and Medicine 2018; Larson et al. 2019; Moucheraud et al. 2022), so we look at quality of care in different ways.

- Perceived treatment index, which captures domains of nonjudgmental, respectful care: average value across 29 items (see Online Appendix Box 1) that can range from 1 to 4. Items were drawn from validated scales measuring quality in family planning care (Sudhinaraset et al. 2018; Jain et al. 2019; Holt et al. 2019; Dehlendorf et al. 2018).
- Perceived person‐centeredness of family planning provider interaction (includes verbal communication, nonverbal communication, and perceived disrespect and abuse (PDA)): average value across 11 items (see Online Appendix Box 2) that can range from 1 to 4.
- Whether the client would recommend the facility to a friend who needed contraceptive services: dichotomous (completely, vs. mostly/somewhat/not at all).

We include quality as an outcome predicted by visit fulfillment, but we acknowledge that this relationship could be bidirectional. We are not trying to draw any causal conclusions from this analysis, and these analyses are purely correlational: we are asking the question “did care received by those whose visit objectives were fulfilled differ from those whose visit objectives were not fulfilled?” Thus, we would draw the same conclusions about the relationship between care quality and fulfillment regardless of which variable is used as the dependent (or independent) variable in our regressions. We chose to use quality measures as the outcome variable because it is more intuitive and interpretable to present the difference in quality between two groups (objective fulfilled vs. objective not fulfilled) than to present the probability of fulfillment across the entire range of continuous quality measures.

### Covariates

We included client's age, marital status, parity, highest educational attainment, and perceived socioeconomic status. We measured perceived socioeconomic status using the following question: “Imagine six steps, where on the bottom, the first step, stand the poorest people, and on the highest step, the sixth, stand the rich. On which step are you today?” (Howe et al. 2011). We grouped clients into three categories: lowest perceived socioeconomic status (steps 1 and 2), middle socioeconomic status (steps 3 and 4), and highest socioeconomic status (steps 5 and 6).

### Analyses

All analyses were conducted using Stata v15. We use generalized bivariable logistic models to assess whether client characteristics hypothesized to be associated with care quality (age, marital status, parity, socioeconomic status, and educational attainment) were associated with visit objective fulfillment (Solo and Festin 2019; Afulani et al. 2021; Dieci et al. 2021; Wollum, Moucheraud, Gipson, et al. 2024); and generalized multivariable linear models to assess the relationship between visit objective fulfillment and care quality with these client characteristics all included as covariates. All models used facility‐level fixed effects to control for differences between facilities. All analyses were conducted separately by country. We controlled for intervention treatment status in all adjusted models.

### Ethical Review

The Beyond Bias study was reviewed and approved by RAND's Human Subjects Protection Committee and ethics review committees in all three countries. This analysis of deidentified secondary data was reviewed by the UCLA IRB, and determined to not be human subjects research.

## RESULTS

Characteristics of the sample are shown in Table 3. Most clients in each country were aged 25 or older, married, did not finish secondary schooling, and in Burkina Faso and Tanzania perceived themselves to be a middle socioeconomic class. Most clients had at least one child (many had three or more, especially in Burkina Faso and Pakistan); and in Burkina Faso and Tanzania, most clients were seeking birth spacing, while in Pakistan 40 percent of clients did not want any more children (this was reported by <15 percent of respondents in Burkina Faso and Tanzania).

**TABLE 3:** Characteristics of the Sample (total *n* =71,602), *n* (%)

	Burkina Faso (*n* = 39,805)	Pakistan (*n* = 7661)	Tanzania (*n* = 24,136)
Age			
≤ 19 years	4171 (10.5%)	677 (8.8%)	1604 (6.6%)
20‐24 years	11,468 (28.8%)	3110 (40.6%)	8576 (35.5%)
≥ 25 years	24,166 (60.7%)	3874 (50.6%)	13,956 (57.8%)
Marital status			
Single	3743 (9.4%)	19 (0.2%)	693 (2.9%)
In a relationship, not living together	2359 (5.9%)	2 (0%)	3337 (13.8%)
In a relationship, living together	2764 (6.9%)	0 (0%)	3967 (16.4%)
Married	30,933 (77.7%)	7637 (99.7%)	16,120 (66.8%)
Educational attainment			
No education	17,652 (44.3%)	2223 (29.0%)	760 (3.1%)
Primary	9723 (24.4%)	2195 (28.7%)	12,686 (52.6%)
Secondary or more	12,430 (31.2%)	3243 (42.3%)	10,690 (44.3%)
Number of children			
None	3410 (8.6%)	143 (1.9%)	1068 (4.4%)
1 child	10,066 (25.3%)	1633 (21.3%)	8731 (36.2%)
2 children	9213 (23.1%)	2376 (31.0%)	7360 (30.5%)
3 or more children	17116 (43.0%)	3509 (45.8%)	6977 (28.9%)
Perceived socioeconomic step^a^			
Lower (steps 1 and 2)	10777 (27.1%)	4105 (53.6%)	7763 (32.2%)
Middle (steps 3 and 4)	26982 (67.8%)	3467 (45.3%)	15455 (64.0%)
Upper (steps 5 and 6)	2046 (5.1%)	89 (1.2%)	918 (3.8%)
How much time until would like to get pregnant			
Do not want another child	4472 (11.7%)	2991 (39.9%)	3237 (14.0%)
Less than 6 months from now	1464 (3.8%)	354 (4.7%)	974 (4.2%)
Between 6 months and a year from now	1607 (4.2%)	480 (6.4%)	1055 (4.6%)
Over a year and less than 5 years from now	16987 (44.3%)	2606 (34.8%)	11064 (47.9%)
Between 5 and 10 years from now	7973 (20.8%)	1048 (14.0%)	5471 (23.7%)
When I get married or after I finish school	5852 (15.3%)	12 (0.2%)	1296 (5.6%)

^a^ Perceived socioeconomic step is from a question asking respondents to choose that step that best represents their relative socioeconomic status within their country.

### Visit Objective

The distribution of visit objectives (and visit outcomes) in each country is shown in Figure 1; the distribution of contraceptive method use (among those already using) by visit objective is shown in Online Appendix Table 1, and the desired method among new users (those intending adoption and restart) is shown in Online Appendix Table 2. In all three countries, method continuation (i.e., wanting to keep the same eligible contraceptive method) and adoption (i.e., wanting to start using an eligible contraceptive method) were the most common visit objectives. In Tanzania, continuation was dominant, reported by 64.1 percent of respondents, and 16.1 percent sought adoption. In Pakistan, approximately equal shares of respondents sought adoption and continuation (44.8 percent and 43.8 percent of respondents, respectively). In Tanzania, 36.2 percent of respondents sought continuation and 29.1 percent sought adoption. In Burkina Faso and Pakistan, approximately 10 percent of respondents intended to restart a method (i.e., had used an eligible contraceptive method in the past and wanted to begin using it again), and in Tanzania, 21.0 percent of respondents sought to restart the method used. Switching from one eligible contraceptive method to another was reported by 5.8 percent of respondents in Burkina Faso, 2.1 percent of respondents in Pakistan, and 10.9 percent of respondents in Tanzania. Seeking discontinuation of an IUD or implant was reported by only 3.6 percent of respondents in Burkina Faso, 0.3 percent of respondents in Pakistan, and 2.8 percent of respondents in Tanzania.

FIGURE 1Visit objectives and outcomes

**Figure:**
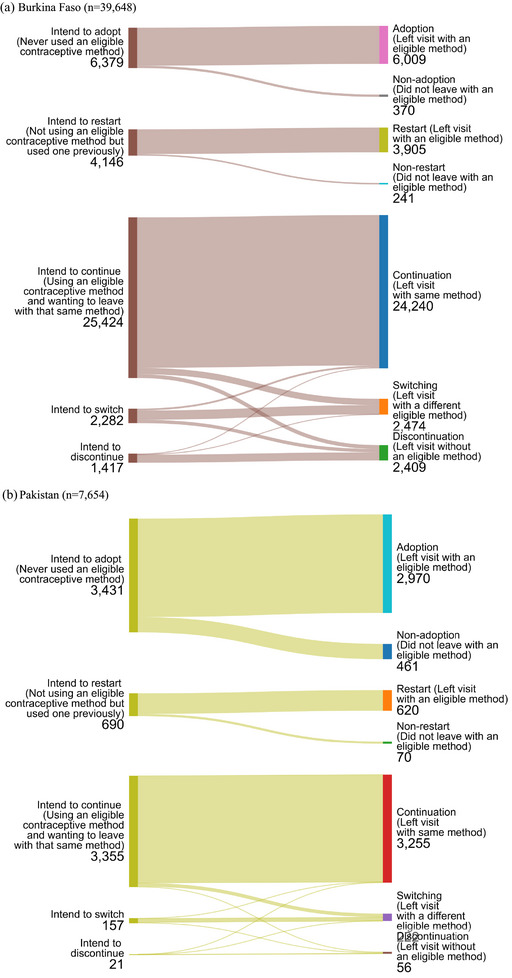

**Figure:**
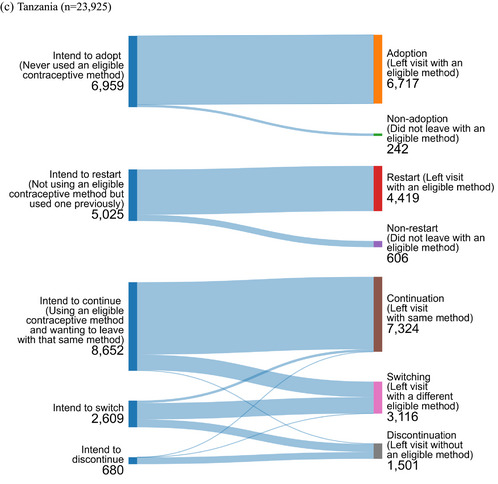

### Visit Objective Fulfillment

Clients seeking a change of method were the group least likely to have their objective met in all three countries: 63.7 percent of clients in Burkina Faso, 73.3 percent in Pakistan, and 61.1 percent in Tanzania who wanted to switch actually achieved this during the visit (Figure 1 and Online Appendix Table 3). In Burkina Faso and Tanzania, between 20 percent and 30 percent of these clients discontinued use (i.e., left the facility without any method), and in all three countries, approximately 10 percent of clients seeking a switch ultimately left the facility with the same method intact or received the same method again (Figure 1 and Online Appendix Table 3).

In all three countries, clients who were not using a method of family planning mostly had their visit objective met: over 85 percent of clients seeking to adopt a method, had their objective fulfilled, as did clients seeking to restart a method (Figure 1 and Online Appendix Table 3).

Over 90 percent of clients seeking continuation in Burkina Faso and Pakistan also achieved their desired outcome (Figure 1 and Online Appendix Table 3). Somewhat fewer (82 percent) clients in Tanzania who wanted to continue their method actually did so, while 18 percent of these Tanzanian clients seeking continuation ultimately switched methods (Figure 1 and Online Appendix Table 3).

In Burkina Faso, 89.3 percent of clients seeking discontinuation had their visit objective met (9.7 percent continued), as did 96.9 percent of clients in Tanzania (Figure 1 and Online Appendix Table 3). In Pakistan, it was a smaller group of clients who sought discontinuation (*n* = 23) and approximately one‐fifth ultimately continued their method (Figure 1 and Online Appendix Table 3).

In Burkina Faso, clients who perceived themselves to be in a lower socioeconomic group were less likely to fulfill their visit objective of switching a method compared to those in higher socioeconomic groups (unadjusted odds ratios displayed in Online Appendix Table 4). In Tanzania those aged 25 and over were less likely than younger clients to receive their desired switch (Online Appendix Table 4). Clients, who had a secondary education and beyond in Burkina Faso, were more likely than clients with no formal education to have their visit objective to switch fulfilled (Online Appendix Table 4). In Burkina Faso and Tanzania, there was a strong parity gradient for achieving a desired switch and a desired restart, with nulliparous clients least likely to have these visit objectives met and those with ≥3 children most likely (Online Appendix Table 4).

### Method Preference Fulfillment

In Tanzania, visit objective fulfillment was lower among clients who did not have a clear method preference than among those with a preferred method (Online Appendix Table 5). There were no clear patterns of difference in counseling methods by visit objective fulfillment (Online Appendix Table 6). In all three countries, a larger percentage of clients who had their visit objective met said that the provider had asked about their method preference compared to clients whose visit objective was not met and this difference was largest in Tanzania (Online Appendix Table 7): 89.2 percent versus 83.0 percent in Burkina Faso, 80.9 percent versus 79.3 percent in Pakistan, and 94.9 percent versus 82.8 percent in Tanzania.

In Burkina Faso, approximately 90 percent of clients seeking to adopt a method received their preferred method (Online Appendix Figure 1). In Pakistan, although the sample size was small, method preference fulfillment among clients seeking to adopt a method was similarly high for all methods except implants: only half of those seeking to adopt an implant received one (Online Appendix Figure 1). In Tanzania, nearly all clients (97.2 percent) who wanted to adopt an implant did so and 89.7 percent of those wanting an IUD inserted received one—but lower percentage of clients seeking injections and pills received these (79.3 percent and 83.8 percent, respectively) (Online Appendix Figure 1). Most of these Tanzanian clients with an unfulfilled desire to adopt an injection or pill were actually given an implant (Online Appendix Figure 1).

A very similar pattern was seen for those seeking to restart a method: in Burkina Faso, most were able to restart their method of choice, as were most in Pakistan except those who wanted to restart implants (many of these clients received an injection instead) (Online Appendix Figure 1). In Tanzania, approximately 20–25 percent of clients who wanted to restart an injection or the pill were instead given an implant (Online Appendix Figure 1).

In Burkina Faso and Tanzania, those who were using injections and pills were most likely to be switched to implants (approximately 38–48 percent in Burkina Faso, and 74–80 percent in Tanzania); although some clients in both countries seeking to switch from injections went to pills (21 percent in Burkina Faso and 13 percent in Tanzania), and some seeking to switch from pills went to injections (42 percent in Burkina Faso and 17 percent in Tanzania) (data not shown). In Burkina Faso, these patterns of switching were largely aligned with client method preferences, with the exception of those who did not receive a method (9–10 percent of those who had a preference to switch to an implant, injection, IUD, or pill continued their method; Online Appendix Figure 1). In Tanzania, nearly all clients seeking to switch to an implant received one (96 percent) while among those who wanted to switch to an injection, 77 percent received an injection and 5 percent received an implant, 5 percent received pills, and 13 percent continued use of the same method.

Clients seeking to receive another implant (continue) were largely able to do so, but approximately 20–30 percent of these clients in each country either received no method or kept the same implant; similarly, half of those seeking to continue their IUD in Burkina Faso were discontinued, as were 28 percent of those in Pakistan and 34 percent of those in Tanzania (Online Appendix Figure 1). Clients who wanted to continue injections and pills were largely able to do so, although, in Tanzania, approximately 12 percent in each of these groups were switched to implants (Online Appendix Figure 1).

### Quality and Visit Objective Fulfillment

**FIGURE 2:**
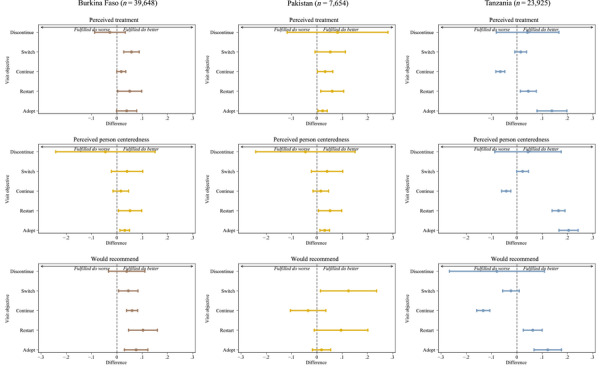
Reported quality of care by visit objective fulfillment (adjusted odds ratios and 95 percent confidence intervals) NOTE: Figures represent predicted differences between those who had their objective fulfilled and those who did not have their objective fulfilled using a fixed effects regression controlling for Beyond Bias intervention status, client age, marital status, parity, education, and perceived socioeconomic status.

Clients in all three countries whose objective to adopt or restart was met reported significantly better perceived treatment than those whose objective to adopt or restart was not met (Figure 2). Perceived person‐centeredness of family planning provider interaction was also higher among clients with a fulfilled objective to adopt or restart in all three countries. Clients were significantly more likely to say they would recommend the facility to a friend when their visit objective to adopt, restart, continue, or switch was fulfilled as in Burkina Faso; in Pakistan, when their objective to switch was fulfilled; and in Tanzania when their objective to adopt or restart was fulfilled. (Clients in Tanzania who had their visit objective to continue fulfilled were paradoxically less likely to say they would recommend the facility to a friend.)

## DISCUSSION

In this large, three‐country analysis, we document the visit objectives of women seeking contraceptive care and the extent to which these objectives were fulfilled. We find that between one‐fifth and one‐third of clients in Burkina Faso and Tanzania who wanted to switch methods (usually from an implant or an IUD) actually left without a method or left with their same method intact. A recent study in Burkina Faso and Kenya likewise found that approximately 15 percent of women using implants did not succeed in obtaining the desired removal (Tumlinson et al. 2023; Wollum, Moucheraud, Sabasaba, et al. 2024). More research is needed to understand why this is occurring. One reason may be stockouts of family planning commodities, which could leave clinicians incapable of meeting clients’ needs; however, this should have affected method take‐up across all visit objective types, which was not the case. Additionally, during the period of this study, family planning commodity stockouts were reportedly rare: in a survey of family planning clinicians at these same facilities around the same time, 97 percent of those in Tanzania said there were no recent stockouts of implants and 99 percent said there were no recent stockouts of IUDs, and in Burkina Faso, 75 percent reported no recent stockouts of implants and 87 percent reported no recent stockouts of IUDs (Wagner et al. 2023b). Although more providers in Pakistan reported recent implant and IUD stockouts [48 percent and 41 percent, respectively] (Wagner et al. 2023b), more implant and IUD users seeking to switch methods had their preference fulfilled in our client data from Pakistan. Understanding provider‐level factors associated with a lack of method switching is an important area for future research. Removal of these methods requires trained and skilled providers as well as resources like ultrasound, which may be limited in some contexts (Prine and Shah 2018; Christofield and Lacoste 2016; Senderowicz et al. 2022). Additionally, how these challenges manifest at the provider and client levels—for example, counseling clients to weather side effects, deterring clients from removal due to associated costs, or lack of confidence in removal techniques—is not well‐understood (Yirgu et al. 2020; Callahan et al. 2020; Senderowicz and Kolenda 2022; Sokol et al. 2024).

Many people in each country were visiting a health facility in order to continue their method of family planning: 64 percent of clients in Burkina Faso, 43 percent in Pakistan, and 36 percent in Tanzania reported this as the reason for their visit. There is a need to better understand the care needs of those seeking method continuation and to develop quality indicators for this group (Wollum et al. 2023; Senderowicz et al. 2023). The conceptualization of quality for clients seeking to continue their method is challenging; if a client says they want to keep their same method, would simply fulfilling their request constitute high‐quality care, or should some counseling occur and if so, how much and what should it include? Similarly, if someone wants to discontinue a method, how much counseling should be provided? Extensive clinician questioning and counseling might suggest that the client's preference is less important than the clinician's “expert opinion,” which can detract from their autonomy and decision‐making power.

It is noteworthy that almost 20 percent of clients in Tanzania who wanted to continue their method ultimately switched methods, mostly by moving from injections and pills to implants. It is important to better understand why clients are being shifted to the use of implants, and whether structural factors like global partner and donor enthusiasm for (and close monitoring of) LARC use, LARC‐focused programming, or provider preferences, underlie this tendency, as has been shown in other studies (Senderowicz 2019; Senderowicz et al. 2021). There are qualitative findings that indicate enthusiasm for implants among family planning providers in Tanzania, due to what they perceive as disadvantages of injections and pills; in particular, they believe that women have a quicker return to fertility after discontinuing implants than other hormonal methods (Wagner et al. 2023a). Many clients in all three countries sought a method switch during their visit, and many did not have this objective met. Better understanding of the desires and needs of people seeking a switch, and how family planning services can best serve these people, is essential.

We examined visit objective fulfillment as a possible driver of the family planning care experience. This is a unique contribution to the literature, as very few previous studies have explicitly measured what clients were seeking in their family planning visit although there is some evidence that fulfillment of contraceptive preference may be associated with better outcomes for women—namely, increased method continuation and more consistent method use (Burke and Potter 2023; Cardona, Bishai, and Anglewicz 2024). We find that women who did not have their objectives met reported less positive experiences at the clinic and less person‐centered care. We do, however, acknowledge that visit objective fulfillment is an imperfect outcome, and in some circumstances, not having an objective met could be a good thing. There is extensive guidance on method counseling precisely because people may not have adequate information for well‐formed preferences. However, we find heterogeneity in objective fulfillment. If some clients are “better” at having their preferences met—or if providers are more likely to fulfill certain types of people's preferences—than others, this may represent a disparity in care quality. These results suggest that clients of lower socioeconomic standing in Burkina Faso, younger age in Burkina Faso, less educational attainment in Burkina Faso and Pakistan, and nulliparous women in Burkina Faso and Tanzania (and parous women in Pakistan) may face particular challenges in achieving desired visit outcomes. A recent multicountry study found that women with greater educational attainment and those seeking methods for limiting births (rather than spacing) were most likely to switch (rather than stop) method use (Sarnak et al. 2023) (although a recent study in Burkina Faso found no such woman‐level differences in achieving a desired implant or IUD removal (Sokol et al. 2024), which suggests support for our finding that there may be important differences in visit objective fulfillment across groups.

This analysis has some limitations that should be noted. First, clients were surveyed cross‐sectionally but contraceptive decisions and actions may be longitudinal—for example, an IUD user might have the method removed during one visit and replaced at a subsequent visit, or might receive information and guidance at one visit and do the replacement at a later visit. We were unable to track women over time, and it is possible that some women ultimately fulfilled their initial visit objective beyond the single data point in this analysis. Second, all data may be subject to reporting or recall bias; in particular, women were approached at the conclusion of their visit and asked to recall what their preference had been before receiving any counseling or care, and this might not be well‐remembered or ‐reported. Related, because quality of care and visit objective (and visit objective fulfillment) were measured simultaneously, we are unable to test the directionality of this relationship—that is, did having one's visit objective met cause one to perceive better quality of care, or did receiving high‐quality care influence one to report that their visit objective was met? There also may have been social desirability bias, as these surveys were conducted at health facilities and people may have been inclined to respond in a way they felt would be more acceptable in this setting (e.g., overstating the quality of care they received to be polite). Third, some people who said they did not receive any service that day were not asked all questions about their visit objective so were excluded from this analysis, and it is possible that this introduced some bias (particularly if women had multiple visits which included information‐only encounters). Fourth, this sample was selected only among people visiting the health facility; those who do not seek services are not included and may have different preferences, visit objectives, and behaviors which are not captured here. Lastly, as noted above, visit objective fulfillment likely includes both clients whose knowledge or preferences changed during the visit as a result of high‐quality care and clients who received low‐quality care. We cannot disentangle these two groups, and, therefore, the measure likely includes both desirable and undesirable unfulfillment.

## CONCLUSIONS

This analysis leverages a unique, large, multicountry dataset to examine people's visit objectives for their family planning care encounter, and whether those objectives were fulfilled. We find that people seeking a change in method were particularly unlikely to achieve this. We also found that visit objective fulfillment was associated with lower perceived quality of care among those seeking to adopt or restart a method in Pakistan and Tanzania. As more people adopt modern contraceptive methods, they will be faced with more frequent and more nuanced decisions about method use. Health systems and healthcare workers must adapt their service provision to accommodate this widening range of client needs. What does high‐quality counseling look like for experienced users? How can services best meet the needs of those who want to switch long‐acting methods, or discontinue use altogether? The global family planning community has long focused on new users, but these results underscore the need for further research, policy, and practice in order to more effectively meet the needs of an increasingly diverse community of contraceptive users globally.

## Supporting information




**Appendix Table 1**: Method used at the start of visit, by visit intention
**Appendix Table 2**: Method desired at start of visit, among those seeking to adopt and restart
**Appendix Table 3**: Visit intentions and outcomes, by country
**Appendix Table 4**: Odds of visit intention fulfillment, by country and client characteristics
**Appendix Table 5**: Distribution of method preferences based on fulfillment of visit objective
**Appendix Table 6**: Methods clients were counseled on based on fulfillment of visit objective
**Appendix Table 7**: Provider asked about preferred method based on fulfillment of visit objective
**Appendix Figure 1**: Method preference fulfillment, by country and visit intention
**Appendix Box 1**: List of perceived treatment index components
**Appendix Box 2**: List of perceived person‐centeredness components

## Data Availability

The data that support the findings of this study are available from the corresponding author upon reasonable request.

## References

[sifp12279-bib-0001] Afulani, Patience A. , Beryl A. Ogolla , Edwina N. Oboke , Linnet Ongeri , Sandra J Weiss , Audrey Lyndon , and Wendy Berry Mendes . 2021. “Understanding Disparities in Person‐Centred Maternity Care: The Potential Role of Provider Implicit and Explicit Bias.” Health Policy and Planning 36 (3): 298–311. 33491086 10.1093/heapol/czaa190 PMC8599771

[sifp12279-bib-0002] Ali, Moazzam , Rachel Folz , and Madeline Farron . 2019. “Expanding Choice and Access in Contraception: An Assessment of Intrauterine Contraception Policies in Low and Middle‐Income Countries.” BMC Public Health 19: 1–6. 31856766 10.1186/s12889-019-8080-7 PMC6924003

[sifp12279-bib-0003] Bertrand, Jane T. , John Ross , Tara M. Sullivan , Karen Hardee , and James D. Shelton . 2020. “Contraceptive Method Mix: Updates and Implications.” Global Health: Science and Practice 8 (4): 666–679. 33361234 10.9745/GHSP-D-20-00229 PMC7784075

[sifp12279-bib-0004] Bhan, Nandita , and Anita Raj . 2021. “From Choice to Agency in Family Planning Services.” The Lancet 398 (10295): 99–101. 10.1016/S0140-6736(21)00990-9 33971154

[sifp12279-bib-0005] Britton, Laura E. , Caitlin R. Williams , Dickens Onyango , Debborah Wambua , and Katherine Tumlinson . 2021. ““When It Comes to Time of Removal, Nothing Is Straightforward”: A Qualitative Study of Experiences With Barriers to Removal of Long‐Acting Reversible Contraception in Western Kenya.” Contraception: X 3: 100063. 33912827 10.1016/j.conx.2021.100063 PMC8063731

[sifp12279-bib-0006] Bryson, Amanda , Atsuko Koyama , and Areej Hassan . 2021. “Addressing Long‐Acting Reversible Contraception Access, Bias, and Coercion: Supporting Adolescent and Young Adult Reproductive Autonomy.” Current Opinion in Pediatrics 33 (4): 345–353. 33797464 10.1097/MOP.0000000000001008

[sifp12279-bib-0007] Bullington, Brooke W. , Nathalie Sawadogo , Katherine Tumlinson , Ana Langer , Abdramane Soura , Pascal Zabre , Ali Sie , and Leigh Senderowicz . 2023. “Prevalence of Non‐Preferred Family Planning Methods Among Reproductive‐Aged Women in Burkina Faso: Results From a Cross‐Sectional, Population‐Based Study.” Sexual and Reproductive Health Matters 31 (1): 2174244. 37195714 10.1080/26410397.2023.2174244 PMC10193871

[sifp12279-bib-0008] Burke, Kristen Lagasse , and Joseph E. Potter . 2023. “Meeting Preferences for Specific Contraceptive Methods: An Overdue Indicator.” Studies in Family Planning 54 (1): 281–300. 36705876 10.1111/sifp.12218

[sifp12279-bib-0009] Callahan, Rebecca , Elena Lebetkin , Claire Brennan , Emmanuel Kuffour , Angela Boateng , Samuel Tagoe , Anne Coolen , Mario Chen , Patrick Aboagye , and Aurélie Brunie . 2020. “What Goes in Must Come Out: A Mixed‐Method Study of Access to Contraceptive Implant Removal Services in Ghana.” Global Health: Science and Practice 8 (2): 220–238. 32606092 10.9745/GHSP-D-20-00013 PMC7326509

[sifp12279-bib-0010] Cardona, Carolina , David Bishai , and Philip Anglewicz . 2024. “Does the Fulfillment of Contraceptive Method Preferences Affect Contraceptive Continuation? Evidence From Urban Kenya, Nigeria, and Senegal.” Demographic Research 50: 131–170.

[sifp12279-bib-0011] Christofield, Megan , and Maryjane Lacoste . 2016. “Accessible Contraceptive Implant Removal Services: An Essential Element of Quality Service Delivery and Scale‐Up.” Global Health: Science and Practice 4 (3): 366–372. 27577239 10.9745/GHSP-D-16-00096 PMC5042693

[sifp12279-bib-0012] Camber Collective . 2022. Beyond Bias: Provider Survey & Segmentation Findings. Camber Collective Report.

[sifp12279-bib-0013] Dehlendorf, Christine , Jillian T. Henderson , Eric Vittinghoff , Jody Steinauer , and Danielle Hessler . 2018. “Development of a Patient‐Reported Measure of the Interpersonal Quality of Family Planning Care.” Contraception 97 (1): 34–40. 28935217 10.1016/j.contraception.2017.09.005

[sifp12279-bib-0014] Dieci, Maria , Zachary Wagner , Willa Friedman , Sarah Burgess , Jessica Vandermark , Sandra I. McCoy , Manisha Shah , and William H. Dow . 2021. “Measuring Family Planning Provider Bias: A Discrete Choice Experiment Among Burkinabé, Pakistani, and Tanzanian Providers.” Studies in Family Planning 52 (3): 299–320. 34472623 10.1111/sifp.12170 PMC12063291

[sifp12279-bib-0015] Fabic, Madeleine Short . 2022. “What Do We Demand? Responding to the Call for Precision and Definitional Agreement in Family Planning's “Demand” and “Need” Jargon.” Global Health: Science and Practice 10 (1): e2200030. 35294394 10.9745/GHSP-D-22-00030 PMC8885353

[sifp12279-bib-0016] Holt, Kelsey , Christine Galavotti , Elizabeth Omoluabi , Sneha Challa , Peter Waiswa , and Jenny Liu . 2023. “Preference‐Aligned Fertility Management as a Person‐Centered Alternative to Contraceptive Use‐Focused Measures.” Studies in Family Planning 54 (1): 301–308. 36723038 10.1111/sifp.12228

[sifp12279-bib-0017] Holt, Kelsey , Icela Zavala , Ximena Quintero , Danielle Hessler , and Ana Langer . 2019. “Development and Validation of the Client‐Reported Quality of Contraceptive Counseling Scale to Measure Quality and Fulfillment of Rights in Family Planning Programs.” Studies in Family Planning 50 (2): 137–158. 31120147 10.1111/sifp.12092 PMC6618078

[sifp12279-bib-0018] Howe, Laura D. , James R. Hargreaves , George B. Ploubidis , Bianca L. De Stavola , and Sharon RA Huttly . 2011. “Subjective Measures of Socio‐Economic Position and the Wealth Index: A Comparative Analysis.” Health Policy and Planning 26 (3): 223–232. 20817696 10.1093/heapol/czq043

[sifp12279-bib-0019] Howett, Rebecca , Alida M. Gertz , Tiroyaone Kgaswanyane , Gregory Petro , Lesego Mokganya , Sifelani Malima , Tshego Maotwe , Melanie Pleaner , and Chelsea Morroni . 2019. “Closing the Gap: Ensuring Access to and Quality of Contraceptive Implant Removal Services is Essential to Rights‐Based Contraceptive Care.” African Journal of Reproductive Health 23 (4): 19–26. 10.29063/ajrh2019/v23i4.3 PMC12570057 32227736

[sifp12279-bib-0020] Institut National de la Statistique et de la Démographie ‐ INSD/Burkina Faso, and ICF International . 2012. Burkina Faso Enquête Démographique et de Santé et à Indicateurs Multiples (EDSBF‐MICS IV) 2010. Institut National de la Statistique et de la Démographie ‐ INSD/Burkina Faso and ICF International Calverton, MD. http://dhsprogram.com/pubs/pdf/FR256/FR256.pdf.

[sifp12279-bib-0021] Jacinto, Ana , Adalgisa Viola Ronda , Connie Lee , Fariyal F. Fikree , and Eric Ramirez‐Ferrero . 2022. “Introducing Long‐Acting Contraceptive Removal Indicators in a Pilot Study in Mozambique: Dynamics of Discontinuation and Implications for Quality of Care.” Global Health: Science and Practice. 10 (1): e2100252. 35040804 10.9745/GHSP-D-21-00252 PMC8885348

[sifp12279-bib-0022] Jain, Aparna , Kumudha Aruldas , Arupendra Mozumdar , Elizabeth Tobey , and Rajib Acharya . 2019. “Validation of Two Quality of Care Measures: Results From a Longitudinal Study of Reversible Contraceptive Users in India.” Studies in Family Planning 50 (2): 179–193. 31120148 10.1111/sifp.12093

[sifp12279-bib-0023] Larson, Elysia , Jigyasa Sharma , Meghan A. Bohren , and Özge Tunçalp . 2019. “When the Patient Is the Expert: Measuring Patient Experience and Satisfaction With Care.” Bulletin of the World Health Organization 97 (8): 563. 31384074 10.2471/BLT.18.225201 PMC6653815

[sifp12279-bib-0024] Ministry of Health, Community Development, Gender, Elderly, Children ‐ MoHCDGEC/Tanzania Mainland, Ministry of Health ‐ MoH/Zanzibar, National Bureau of Statistics ‐ NBS/Tanzania, Office of Chief Government Statistician ‐ OCGS/Zanzibar, and ICF . 2016. Tanzania Demographic and Health Survey and Malaria Indicator Survey 2015–2016. MoHCDGEC, MoH, NBS, OCGS, and ICF (Dar es Salaam, Tanzania). http://dhsprogram.com/pubs/pdf/FR321/FR321.pdf.

[sifp12279-bib-0025] Moucheraud, Corrina , Kaitlyn McBride , Patrick Heuveline , and Manisha Shah . 2022. “Preventing, but Not Caring for Adolescent Pregnancies? Disparities in the Quality of Reproductive Health Care in Sub‐Saharan Africa.” Journal of Adolescent Health 71 (2): 210–216. 10.1016/j.jadohealth.2022.02.012 PMC9995166 35437221

[sifp12279-bib-0026] Murithi, Lydia , Theo Gibbs , and Rebecca Hope . 2022. “Integrating Human‐Centered Design in a Multidisciplinary Effort to Address Provider Bias: The Beyond Bias Experience.” Beri, Beyond Bias, Camber Collective, Pathfinder, and Y Labs.

[sifp12279-bib-0027] Murithi, Lydia , Sakina Zaidi , Jessica Vandermark , and Mohamed Brooks . 2021. Integrating Segmentation Analysis Into Sexual and Reproductive Health and Rights Programs. Watertown, MA: Pathfinder International.

[sifp12279-bib-0028] National Academies of Sciences, Engineering, and Medicine . 2018. Crossing the Global Quality Chasm: Improving Health Care Worldwide. Washington, DC: The National Academies Press. 30605296

[sifp12279-bib-0029] National Institute of Population Studies ‐ NIPS/Pakistan, and ICF . 2019. “Pakistan Demographic and Health Survey 2017–18.” Islamabad, Pakistan: NIPS/Pakistan and ICF. http://dhsprogram.com/pubs/pdf/FR354/FR354.pdf.

[sifp12279-bib-0030] United Nations . 2022. “World Population Prospects: The 2022 Revision.” Population Division Department of Economic and Social Affairs, United Nations.

[sifp12279-bib-0031] Pathfinder International, Camber Collective, BERI, YLabs . 2019. “Beyond Bias: Project Overview.” Washington, DC: Pathfinder.

[sifp12279-bib-0032] Prine, Linda , and Meera Shah . 2018. “Long‐Acting Reversible Contraception: Difficult Insertions and Removals.” American Family Physician 98 (5): 304–309. 30216029

[sifp12279-bib-0033] Sarnak, Dana , Alison Gemmill , Sarah EK Bradley , Eve Brecker , and Kaitlyn Patierno . 2023. “Stop or Switch: Correlates of Stopping Use or Switching Contraceptive Methods While Wanting to Avoid Pregnancy in 48 Low‐and Middle‐Income Countries.” *Studies in Family Planning*. 10.1111/sifp.12221 36723513

[sifp12279-bib-0034] Senderowicz, Leigh. 2019. ““I Was Obligated to Accept”: A Qualitative Exploration of Contraceptive Coercion.” Social Science & Medicine 239: 112531. 31513932 10.1016/j.socscimed.2019.112531

[sifp12279-bib-0035] Senderowicz, Leigh. 2020. “Contraceptive Autonomy: Conceptions and Measurement of a Novel Family Planning Indicator.” Studies in Family Planning 51 (2): 161–176. 32358789 10.1111/sifp.12114

[sifp12279-bib-0036] Senderowicz, Leigh , Brooke W. Bullington , Nathalie Sawadogo , Katherine Tumlinson , Ana Langer , Abdramane Soura , Pascal Zabré , and Ali Sié . 2023. “Measuring Contraceptive Autonomy at Two Sites in Burkina Faso: A First Attempt to Measure a Novel Family Planning Indicator.” Studies in Family Planning 54 (1): 201–230. 36729070 10.1111/sifp.12224 PMC10184300

[sifp12279-bib-0037] Senderowicz, Leigh , Celia Karp , Brooke W. Bullington , Katherine Tumlinson , Linnea Zimmerman , Funmilola M. OlaOlorun , Musa Sani Zakirai , and Performance Monitoring for Action Principal Investigators Group . 2022. “Facility Readiness to Remove Subdermal Contraceptive Implants in 6 Sub‐Saharan African Countries.” AJOG Global Reports 2 (4): 100132. 36444203 10.1016/j.xagr.2022.100132 PMC9700317

[sifp12279-bib-0038] Senderowicz, Leigh , and Al Kolenda . 2022. ““She Told Me No, That You Cannot Change”: Understanding Provider Refusal to Remove Contraceptive Implants.” SSM‐Qualitative Research in Health 2: 100154. 37304900 10.1016/j.ssmqr.2022.100154 PMC10257102

[sifp12279-bib-0039] Senderowicz, Leigh , Erin Pearson , Kristy Hackett , Sarah Huber‐Krum , Joel Msafiri Francis , Nzovu Ulenga , and Till Bärnighausen . 2021. “‘I Haven't Heard Much About Other Methods’: Quality of Care and Person‐Centredness in a Programme to Promote the Postpartum Intrauterine Device in Tanzania.” BMJ Global Health 6 (6): e005775. 10.1136/bmjgh-2021-005775 PMC8230964 34162627

[sifp12279-bib-0040] Sennott, Christie , and Sara Yeatman . 2012. “Stability and Change in Fertility Preferences Among Young Women in Malawi.” International Perspectives on Sexual and Reproductive Health 38 (1): 34. 22481147 10.1363/3803412 PMC3322634

[sifp12279-bib-0041] Sergison, Jill E. , Randy M. Stalter , Rebecca L. Callahan , Kate H. Rademacher , and Markus J. Steiner . 2017. “Cost of Contraceptive Implant Removal Services Must Be Considered When Responding to the Growing Demand for Removals.” Global Health: Science and Practice 5 (2): 330–332. 28655806 10.9745/GHSP-D-17-00100 PMC5487094

[sifp12279-bib-0042] Sokol, Natasha A. , Nathalie Sawadogo , Brooke W. Bullington , Katherine Tumlinson , Ana Langer , Abdramane Soura , Pascal Zabre , Ali Sie , Janet A. Johnson , and Leigh Senderowicz . 2024. “Perceptions of Access to Long‐Acting Reversible Contraception Removal Among Women in Burkina Faso.” Contraception 129: 110302. 37802461 10.1016/j.contraception.2023.110302 PMC11285006

[sifp12279-bib-0043] Solo, Julie , and Mario Festin . 2019. “Provider Bias in Family Planning Services: A Review of Its Meaning and Manifestations.” Global Health: Science and Practice 7 (3): 371–385. 31515240 10.9745/GHSP-D-19-00130 PMC6816811

[sifp12279-bib-0044] Speizer, Ilene S. , Jason Bremner , and Shiza Farid . 2022. “Language and Measurement of Contraceptive Need and Making These Indicators More Meaningful for Measuring Fertility Intentions of Women and Girls.” Global Health: Science and Practice 10 (1): e2100450. 35294385 10.9745/GHSP-D-21-00450 PMC8885354

[sifp12279-bib-0045] Starrs, Ann M. , Alex C. Ezeh , Gary Barker , Alaka Basu , Jane T. Bertrand , Robert Blum , Awa M. Coll‐Seck , Anand Grover , Laura Laski , and Monica Roa . 2018. “Accelerate Progress—Sexual and Reproductive Health and Rights for All: Report of the Guttmacher–Lancet Commission.” The Lancet 391 (10140): 2642–2692. 10.1016/S0140-6736(18)30293-9 29753597

[sifp12279-bib-0046] Sudhinaraset, May , Patience A. Afulani , Nadia Diamond‐Smith , Ginger Golub , and Aradhana Srivastava . 2018. “Development of a Person‐Centered Family Planning Scale in India and Kenya.” Studies in Family Planning 49 (3): 237–258. 30069983 10.1111/sifp.12069

[sifp12279-bib-0047] Trinitapoli, Jenny , and Sara Yeatman . 2018. “The Flexibility of Fertility Preferences in a Context of Uncertainty.” Population and Development Review 44 (1): 87. 29695890 10.1111/padr.12114 PMC5900734

[sifp12279-bib-0048] Tumlinson, Katherine , Leigh Senderowicz , Brooke W. Bullington , Stephanie Chung , Emilia Goland , Linnea Zimmerman , Peter Gichangi , Mary Thiongo , Georges Guiella , and Celia Karp . 2023. “Assessing Trends and Reasons for Unsuccessful Implant Discontinuation in Burkina Faso and Kenya Between 2016 and 2020: A Cross‐Sectional Study.” BMJ Open 13 (7): e071775. 10.1136/bmjopen-2023-071775 PMC10357675 37463804

[sifp12279-bib-0049] United Nations . 2022. “Model‐Based Estimates and Projections of Family Planning Indicators. edited by Department of Economic and Social Affairs Population Division.” New York: United Nations Department of Economic and Social Affairs, Population Division.

[sifp12279-bib-0050] United Nations Department of Economic and Social Affairs . 2020. World Fertility and Family Planning 2020. New York: United Nations Department of Economic and Social Affairs, Population Division.

[sifp12279-bib-0052] Wagner, Zachary , Corrina Moucheraud , Manisha Shah , Alexandra Wollum , Willa H. Friedman , and William H. Dow . 2023a. “Reducing Bias Among Health Care Providers: Experimental Evidence From Tanzania, Burkina Faso, and Pakistan.” Cambridge, MA: National Bureau of Economic Research.

[sifp12279-bib-0053] Wagner, Zachary , Corrina Moucheraud , Manisha Shah , Alexandra Wollum , Willa H. Friedman , and William H. Dow . 2023b. “Beyond Bias Provider Survey Data.” Harvard Dataverse.

[sifp12279-bib-0054] Wollum A. , Moucheraud C. , Wagner Z. , Gipson J. . 2023. “Removals of Long‐Acting Reversible Contraceptive Methods and Quality of Care in Tanzania: Client and Provider Perspectives.” Paper Presented at Population Association of America Annual Meeting, New Orleans, LA.

[sifp12279-bib-0055] Wollum, Alexandra , Corrina Moucheraud , Jessica D. Gipson , Willa Friedman , Manisha Shah , and Zachary Wagner . 2024. “Characterizing Provider Bias in Contraceptive Care in Tanzania and Burkina Faso: A Mixed‐Methods Study.” Social Science & Medicine 348: 116826. 38581812 10.1016/j.socscimed.2024.116826

[sifp12279-bib-0056] Wollum, Alexandra , Corrina Moucheraud , Amon Sabasaba , and Jessica D. Gipson . 2024. “Removal of Long‐Acting Reversible Contraceptive Methods and Quality of Care in Dar es Salaam, Tanzania: Client and Provider Perspectives From a Secondary Analysis of Cross‐Sectional Survey Data From a Randomized Controlled Trial.” PLoS Global Public Health 4 (1): e0002810. 38261598 10.1371/journal.pgph.0002810 PMC10805313

[sifp12279-bib-0057] World Bank . 2017. “World Development Indicators.” Accessed September 1, 2023. http://databank.worldbank.org/data/home.aspx.

[sifp12279-bib-0058] World Bank . 2020. “Country and Lending Groups.” http://data.worldbank.org/about/country‐and‐lending‐groups.

[sifp12279-bib-0059] Yeatman, Sara , Christie Sennott , and Steven Culpepper . 2013. “Young Women's Dynamic Family Size Preferences in the Context of Transitioning Fertility.” Demography 50 (5): 1715–1737. 23619999 10.1007/s13524-013-0214-4 PMC3786023

[sifp12279-bib-0060] Yirgu, Robel , Shannon N. Wood , Celia Karp , Amy Tsui , and Caroline Moreau . 2020. ““You Better Use the Safer One… Leave This One”: The Role of Health Providers in Women's Pursuit of Their Preferred Family Planning Methods.” BMC Women's Health 20: 1–9. 32787924 10.1186/s12905-020-01034-1 PMC7425019

