# Supplementary material for: Contraceptive Care Visit Objectives and Outcomes: Evidence From Burkina Faso, Pakistan, and Tanzania
Source: Stud Fam Plann. 2024 Dec 3;55(4):315–32. doi: 10.1111/sifp.12279 (PMC11636775; doi:10.1111/sifp.12279)
Supplement: Supplementary file 1 — Appendix Table 1: Method used at the start of visit, by visit intention Appendix Table 2: Method desired at start of visit, among those seeking to adopt and restart Appendix Table 3: Visit intentions and outcomes, by country Appendix Table 4: Odds of visit intention fulfillment, by country and client characteristics Appendix Table 5: Distribution of method preferences based on fulfillment of visit objective Appendix Table 6: Methods clients were counseled on based on fulfillment of visit objective Appendix Table 7: Provider asked about preferred method based on fulfillment of visit objective Appendix Figure 1: Method preference fulfillment, by country and visit intention Appendix Box 1: List of perceived treatment index components Appendix Box 2: List of perceived person‐centeredness components [file SIFP-55-315-s001.docx]

**Appendix Table 1**: Method used at start of visit, by visit intention

|  | **Intention: Switch** | **Intention: Continue** | **Intention: Discontinue** | **Intention: Restart*** |
| --- | --- | --- | --- | --- |
| **Burkina Faso** | n= 2,282 | n= 25,424 | n= 1,417 | n= 4,129 |
| Implant | 1280 (56.1%) | 2659 (10.5%) | 1290 (91%) | 1,707 (41.3%) |
| Injection | 505 (22.1%) | 17993 (70.8%) | 0 | 1,595 (38.6%) |
| IUD | 162 (7.1%) | 699 (2.7%) | 127 (9%) | 267 (6.5%) |
| Pill | 335 (14.7%) | 4073 (16.0%) | 0 | 655 (15.9%) |
| **Pakistan** | n=157 | n=3,328 | n=21 | n=685 |
| Implant | 11 (7%) | 9 (.3%) | 3 (14.3%) | 23 (3.4%) |
| Injection | 45(28.7%) | 2574(76.7%) | 0 | 418 (61.2%) |
| IUD | 37(23.6%) | 67(2%) | 18 (85.7%) | 76 (11.1%) |
| Pill | 64(40.8%) | 705(21.0%) | 0 | 212 (31.0%) |
| **Tanzania** | n= 2,609 | n=8,652 | n= 680 | n= 4,777 |
| Implant | 1448 (55.5%) | 3016 (34.9%) | 649 (95.4%) | 1,945 (40.7%) |
| Injection | 834 (32.0%) | 4113 (47.5%) | 0 | 2,252 (47.1%) |
| IUD | 65 (2.5%) | 77 (.9%) | 31 (4.6%) | 104 (2.2%) |
| Pill | 262 (10%) | 1446 (16.7%) | 0 | 793 (16.6%) |

*Note: Respondents could report more than one method*

**Methods used in past*

*Table excludes people who did not report which method they were using previously.*

**Appendix Table 2**: Method desired at start of visit, among those seeking to adopt and restart

|  | **Burkina Faso** | | **Pakistan** | | **Tanzania** | |
| --- | --- | --- | --- | --- | --- | --- |
|  | Intention: Adopt  (n= 6,379) | Intention: Restart  (n=4,129) | Intention: Adopt  (n= 3,431) | Intention: Restart  (n=681) | Intention: Adopt  (n= 6,967) | Intention: Restart  (n=4,779) |
| Implant | 2,871 (45.0%) | 1484  (35.9%) | 24 (0.7%) | 14 (2.1%) | 3,751 (53.8%) | 1969 (41.2%) |
| Injection | 1,943 (30.5%) | 1565  (37.9%) | 512 (14.9%) | 327 (48.0%) | 1,194 (17.1%) | 1507 (31.5%) |
| IUD | 279 (4.4%) | 388  (9.4%) | 241 (7.0%) | 102 (15.0 %) | 117 (1.7%) | 147 (3.1%) |
| Pill | 495 (7.8%) | 549 (13.3%) | 232 (6.8%) | 87 (12.8%) | 302 (4.3%) | 489 (10.2%) |
| Other/multiple | 104 (1.4%) | 31 (0.8%) | 119 (3.5%) | 66 (9.7%) | 198 (2.8%) | 193 (4.0%) |
| Unsure | 703 (11.0 %) | 112 (2.7%) | 2,303 (67.1%) | 85 (12.5%) | 1,405 (20.2%) | 474 (9.9%) |

**Appendix Table 3**: Visit intentions and outcomes, by country

|  | **Intention: Adopt** | **Intention: Switch** | **Intention: Continue** | **Intention: Discontinue** | **Intention: Restart** |
| --- | --- | --- | --- | --- | --- |
| **Burkina Faso** | n=6,379 | n= 2,282 | n= 25,424 | n= 1,417 | n=4,146 |
| Outcome: Adopt | 6,009 (94.2%) | n/a | n/a | n/a | n/a |
| Outcome: Non-adopt | 370 (5.8%) | n/a | n/a | n/a | n/a |
| Outcome: Switch | n/a | 1,454 (63.7%) | 1,005 (4.0%) | 15 (1.1%) | n/a |
| Outcome: Continue | n/a | 294 (12.9%) | 23,809 (93.7%) | 137 (9.7%) | n/a |
| Outcome: Discontinue | n/a | 534 (23.4%) | 610 (2.4%) | 1,265 (89.3%) | n/a |
| Outcome: Restart | n/a | n/a | n/a | n/a | 3,905 (94.2%) |
| Outcome: Non-restart | n/a | n/a | n/a | n/a | 241 (5.8%) |
| **Pakistan** | n=3,431 | n= 157 | n= 3,355 | n=21 | n=690 |
| Outcome: Adopt | 2,970 (86.6%) | n/a | n/a | n/a | n/a |
| Outcome: Non-adopt | 461 (13.4%) | n/a | n/a | n/a | n/a |
| Outcome: Switch | n/a | 115 (73.3%) | 106 (3.2%) | 1 (4.8%) | n/a |
| Outcome: Continue | n/a | 19 (12.0%) | 3,232 (96.3%) | 4 (19.1%) | n/a |
| Outcome: Discontinue | n/a | 23 (14.7%) | 17 (0.5%) | 16 (76.2%) | n/a |
| Outcome: Restart | n/a | n/a | n/a | n/a | 620 (89.9%) |
| Outcome: Non-restart | n/a | n/a | n/a | n/a | 70 (10.1%) |
| **Tanzania** | n= 6,959 | n= 2,609 | n= 8,652 | n= 680 | n= 5,025 |
| Outcome: Adopt | 6,717 (96.4%) | n/a | n/a | n/a | n/a |
| Outcome: Non-adopt | 242 (3.5%) | n/a | n/a | n/a | n/a |
| Outcome: Switch | n/a | 1,594 (61.1%) | 1,513 (17.5%) | 9 (1.3%) | n/a |
| Outcome: Continue | n/a | 238 (9.1%) | 7,074 (81.8%) | 12 (1.8%) | n/a |
| Outcome: Discontinue | n/a | 777 (29.8%) | 65 (0.8%) | 659 (96.9%) | n/a |
| Outcome: Restart | n/a | n/a | n/a | n/a | 4,419 (87.9%) |
| Outcome: Non-restart | n/a | n/a | n/a | n/a | 606 (12.1%) |

**Appendix Table 4**: Odds of visit intention fulfillment, by country and client characteristics

*p<.05 ** p<.01 *** p<.001

1. Burkina Faso

|  | Adoption intention  (n=6,379) | Switching intention (n=2,282) | Continuation intention  (n= 25,424) | Discontinuation intention  (n=1,417) | Restart intention  (n=4,146) |
| --- | --- | --- | --- | --- | --- |
| **Age** |  |  |  |  |  |
| <=19 | Ref | Ref | Ref | Ref | Ref |
| 20-24 | 1.01 | 1.18 | 1.13 | 1.19 | 1.46 |
| >24 | 0.80 | 1.21 | 1.08 | 1.21 | 1.49 |
| **Marital status** |  |  |  |  |  |
| Not married | Ref | Ref | Ref | Ref | Ref |
| Married | 1.06 | 1.05 | 0.90 | 1.64* | 1.1 |
| **At Least Secondary Education** |  |  |  |  |  |
| No education | Ref | Ref | Ref | Ref | Ref |
| Primary | 1.37 | 1.03 | 1.04 | 1.03 | 1.25 |
| Secondary or more | .81 | 1.27* | 1.00 | 1.16 | 1.04 |
| **Number of children** |  |  |  |  |  |
| No children | Ref | Ref | Ref | Ref | Ref |
| 1 child | 1.47** | 1.13 | 1.02 | 1.56 | 1.71 |
| 2 children | 1.35 | 1.54* | 0.94 | 1.31 | 2.47** |
| 3 or more children | 0.97 | 1.64** | 1.02 | 1.23 | 2.27** |
| **Perceived step (SES)** |  |  |  |  |  |
| 1-2 | Ref | Ref | Ref | Ref | Ref |
| 3-4 | 0.96 | 1.13 | 1.01 | 0.74 | 1.14 |
| 5-6 | 0.87 | 2.60** | 0.96 | 2.38 | 1.57 |
| **Desired spacing** |  |  |  |  |  |
| Less than 6 months | Ref | Ref | Ref | Ref | Ref |
| Between 6 months and a year | 2.09 | 5.80*** | 1.40 | .47** | 3.58** |
| Over a year and less than 5 years | 4.01** | 14.21*** | 1.53* | .23*** | 15.21*** |
| B/w 5 & 10 years | 4.23** | 16.62*** | 1.40 | .19*** | 13.4*** |
| When I get married or after finish school | 3.58** | 9.41*** | 1.37 | .24*** | 12.68*** |
| Do not want another child | 1.89 | 12.06*** | 1.34 | .25*** | 10.53*** |

1. Pakistan

|  | Adoption intention  (n= 3,431) | Switching intention (n= 157) | Continuation intention  (n= 3,355) | Restart intention  (n= 690) |
| --- | --- | --- | --- | --- |
| **Age** |  |  |  |  |
| <=19 | Ref | Ref | Ref | Ref |
| 20-24 | 1.22 | N/A | 1.33 | 1.26 |
| >24 | 1.43 | 2.19 | 0.68 | N/A |
| **Marital status** |  |  |  |  |
| Not married | Ref | N/A | N/A | N/A |
| Married | 1.54 | N/A | N/A | N/A |
| **At Least Secondary Education** |  |  |  |  |
| No education | Ref | Ref | Ref | Ref |
| Primary | .63* | 2.91 | .87 | 2.00 |
| Secondary or more | .68* | 5.35 | 1.02 | 1.00 |
| **Number of children** |  |  |  |  |
| No children | Ref | Ref | Ref | Ref |
| 1 child | 1.03 | .45 | 1.44 | 2.81 |
| 2 children | 1.15 | 1.14 | 1.15 | 0.92 |
| 3 or more children | 1.3 | N/A | N/A | N/A |
| **Perceived step (SES)** |  |  |  |  |
| 1-2 | Ref | Ref | Ref | Ref |
| 3-4 | 1.36* | 2.58 | 0.89 | 0.95 |
| 5-6 | 0.65 | N/A | 0.30 | 0.27 |
| **Desired spacing** |  |  |  |  |
| Less than 6 months | Ref | Ref | Ref | Ref |
| Between 6 months and a year | 1.24 | 0.86 | 1.17 | N/A |
| Over a year and less than 5 years | 0.79 | 1.11 | 1.13 | 24.4* |
| B/w 5 & 10 years | 0.79 | 1.57 | .25* | 10.29 |
| When I get married or after finish school | 0.92 | N/A | N/A | N/A |
| Do not want another child | 0.99 | N/A | .46 | 9.58 |

Note: Those intending to discontinue are not included in this table due to small sample size (n=21)

1. Tanzania

|  | Adoption intention  (n= 6,967) | Switching intention (n= 2,609) | Continuation intention  (n= 8,652) | Discontinuation intention  (n= 680) | Restart intention  (n= 5,025) |
| --- | --- | --- | --- | --- | --- |
| **Age** |  |  |  |  |  |
| <=19 | Ref | Ref | Ref | Ref | Ref |
| 20-24 | 1.06 | 0.64 | 0.50*** | 4.22 | 1.90 |
| >24 | 1.01 | 0.55* | 0.74 | 3.18 | 2.13* |
| **Marital status** |  |  |  |  |  |
| Not married | Ref | Ref | Ref | Ref | Ref |
| Married | 1.24 | 0.95 | 0.91 | 1.61 | 1.28* |
| **At Least Secondary Education** |  |  |  |  |  |
| No education | Ref | Ref | Ref | Ref | Ref |
| Primary | .46 | 1.01 | .87 | .67 | .69 |
| Secondary or more | .60 | .97 | 1.11 | 1.00 | .60 |
| **Number of children** |  |  |  |  |  |
| No children | Ref | Ref | Ref | Ref | Ref |
| 1 child | 1.23 | 1.19 | 0.64 | 1.57 | 1.52 |
| 2 children | 1.98* | 1.68 | 0.69 | 0.99 | 2.16* |
| 3 or more children | 1.45 | 2.02* | 0.81 | 1.12 | 2.66** |
| **Perceived step (SES)** |  |  |  |  |  |
| 1-2 | Ref | Ref | Ref | Ref | Ref |
| 3-4 | 1.29 | 0.87 | 1.38*** | 1.41 | 1.02 |
| 5-6 | 1.00 | 0.98 | 2.1*** | 1.91 | 0.50* |
| **Desired spacing** |  |  |  |  |  |
| Less than 6 months | Ref | Ref | Ref | Ref | Ref |
| Between 6 months and a year | 0.97 | 4.96*** | 1.03 | 0.42 | 6.85*** |
| Over a year and less than 5 years | 4.01* | 17.36*** | 1.06 | 0.05* | 27.94*** |
| B/w 5 & 10 years | 3.03 | 32.3*** | 0.73 | 0.06* | 31.09*** |
| When I get married or after finish school | 5.36* | 9.69*** | 0.97 | 0.18 | 47.02*** |
| Do not want another child | 2.2 | 15.72*** | 0.82 | 0.07* | 23.35*** |

Tables display unadjusted odds ratios from bivariable logistic regressions.

**Appendix Table 5:** Distribution of method preferences based on fulfillment of visit objective

|  | **Burkina Faso** | | **Pakistan** | | **Tanzania** | |
| --- | --- | --- | --- | --- | --- | --- |
|  | Visit objective fulfilled  (N=35,177) | Visit objective not fulfilled  (N=3,032) | Visit objective fulfilled  (N=6,936) | Visit objective not fulfilled  (N=688) | Visit objective fulfilled  (N=19,799) | Visit objective not fulfilled  (N=1,255) |
| Implant | 6959 (19.8%) | 559 (18.4%) | 39 (0.6%) | 11 (1.6%) | 8924 (45.1%) | 663 (20.9%) |
| Injection | 21003 (59.7%) | 1071 (35.3%) | 3364 (48.5%) | 104 (15.1%) | 6358 (32.1%) | 804 (25.3%) |
| IUD | 1451 (4.1%) | 93 (3.1%) | 398 (5.7%) | 47 (6.8%) | 342 (1.7%) | 41 (1.3%) |
| Pill | 4950 (14.1%) | 564 (18.6%) | 974 (14.0%) | 55 (8.0%) | 2122 (10.7%) | 384 (12.1%) |
| Other/multiple | 116 (.3%) | 17 (0.6%) | 151 (2.2%) | 41 (6.0%) | 389 (2.0%) | 30 (.9%) |
| Unsure | 698 (2.1%) | 728 (24.0%) | 2010 (29.0%) | 430 (62.5%) | 1664 (8.4%) | 1255 (39.5%) |

Table includes clients with visit objectives: Adopt, switch, continue, restart

**Appendix Table 6**: Methods clients were counseled on based on fulfillment of visit objective

|  | **Burkina Faso** | | **Pakistan** | | **Tanzania** | |
| --- | --- | --- | --- | --- | --- | --- |
|  | Visit objective fulfilled  (N=36,439) | Visit objective not fulfilled  (N=3,203) | Visit objective fulfilled  (N=6,916) | Visit objective not fulfilled  (N=697) | Visit objective fulfilled  (N=20,454) | Visit objective not fulfilled  (N=3,470) |
| Implant | 26394 (72.4%) | 2382 (74.4%) | 1856 (26.8%) | 302 (43.3%) | 15292 (74.8%) | 2685 (77.4%) |
| Injection | 31446 (86.3%) | 2240 (69.9%) | 5127 (74.0%) | 494 (70.8%) | 13581 (66.4%) | 1704 (49.1%) |
| IUD | 24341 (66.8%) | 1782 (55.6%) | 3177 (45.9%) | 529 (75.8%) | 10909 (53.3%) | 1597 (46.1%) |
| Pill | 25345 (69.6%) | 1999 (62.4%) | 2678 (38.7%) | 398 (56.9%) | 10907 (53.3%) | 1526 (44.0%) |
| Condom | 17971 (49.3%) | 1358 (42.4%) | 1040 (15.0%) | 186 (26.6%) | 7545 (36.9%) | 1193 (34.4%) |
| EC | 7196 (19.7%) | 514 (16.0%) | 406 (5.9%) | 65 (9.3%) | 2483 (12.1%) | 147 (4.2%) |
| Natural | 9473 (26.0%) | 746 (23.3%) | 267 (3.9%) | 43 (6.2%) | 3052 (14.9%) | 179 (5.2%) |

Table includes clients with visit objectives: Adopt, switch, continue, discontinue, restart

**Appendix Table 7**: Provider asked about preferred method based on fulfillment of visit objective

|  | Visit objective fulfilled | Visit objective not fulfilled |
| --- | --- | --- |
| Burkina Faso | 28,164 (89.2%) | 2,102 (83.0%) |
| Pakistan | 4,694 (80.9%) | 479 (79.3%) |
| Tanzania | 16,107 (94.9%) | 2,019 (82.8%) |

**Appendix Figure 1**: Method preference fulfillment, by country and visit intention

Burkina Faso


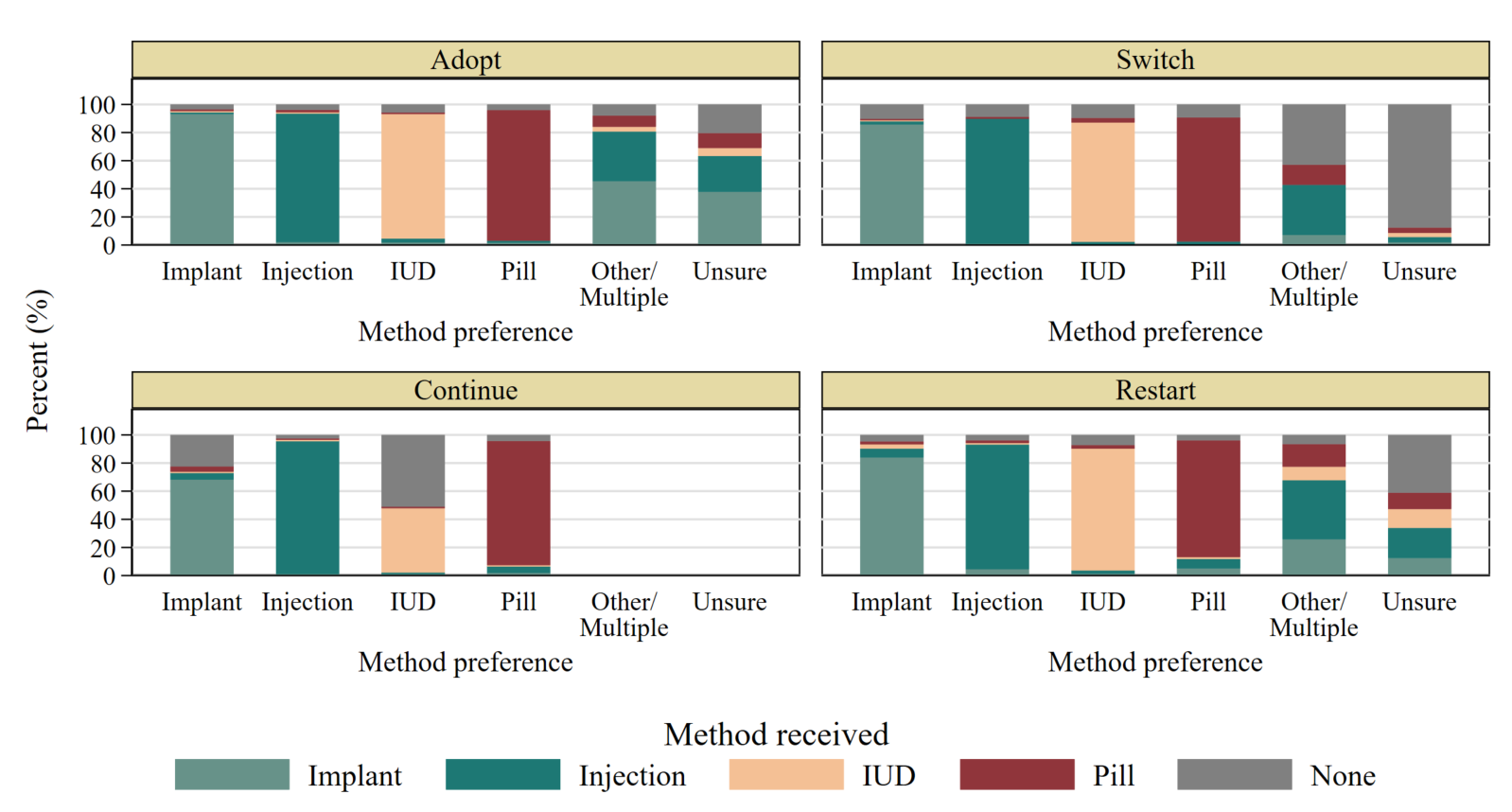


Pakistan


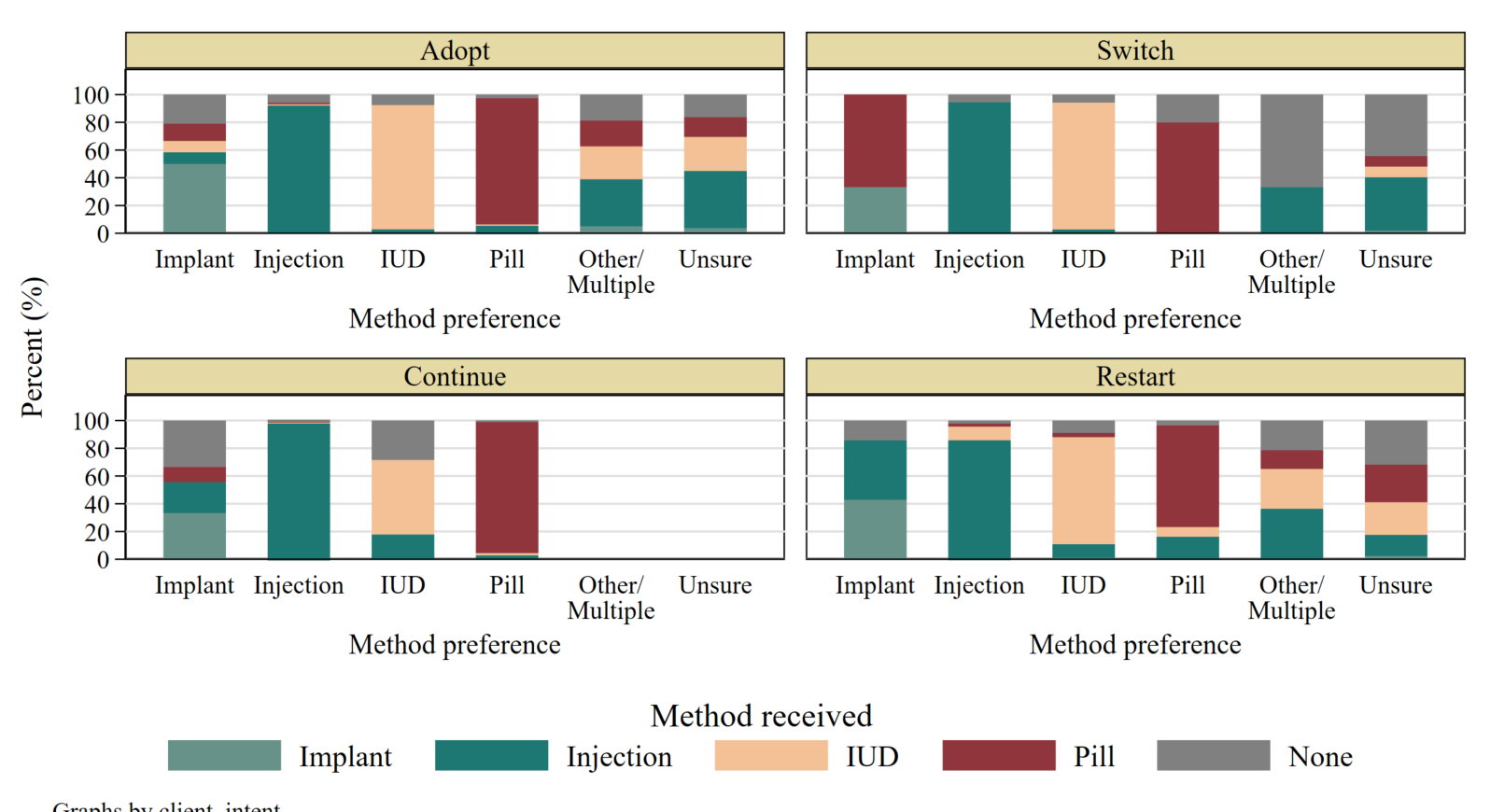


Tanzania


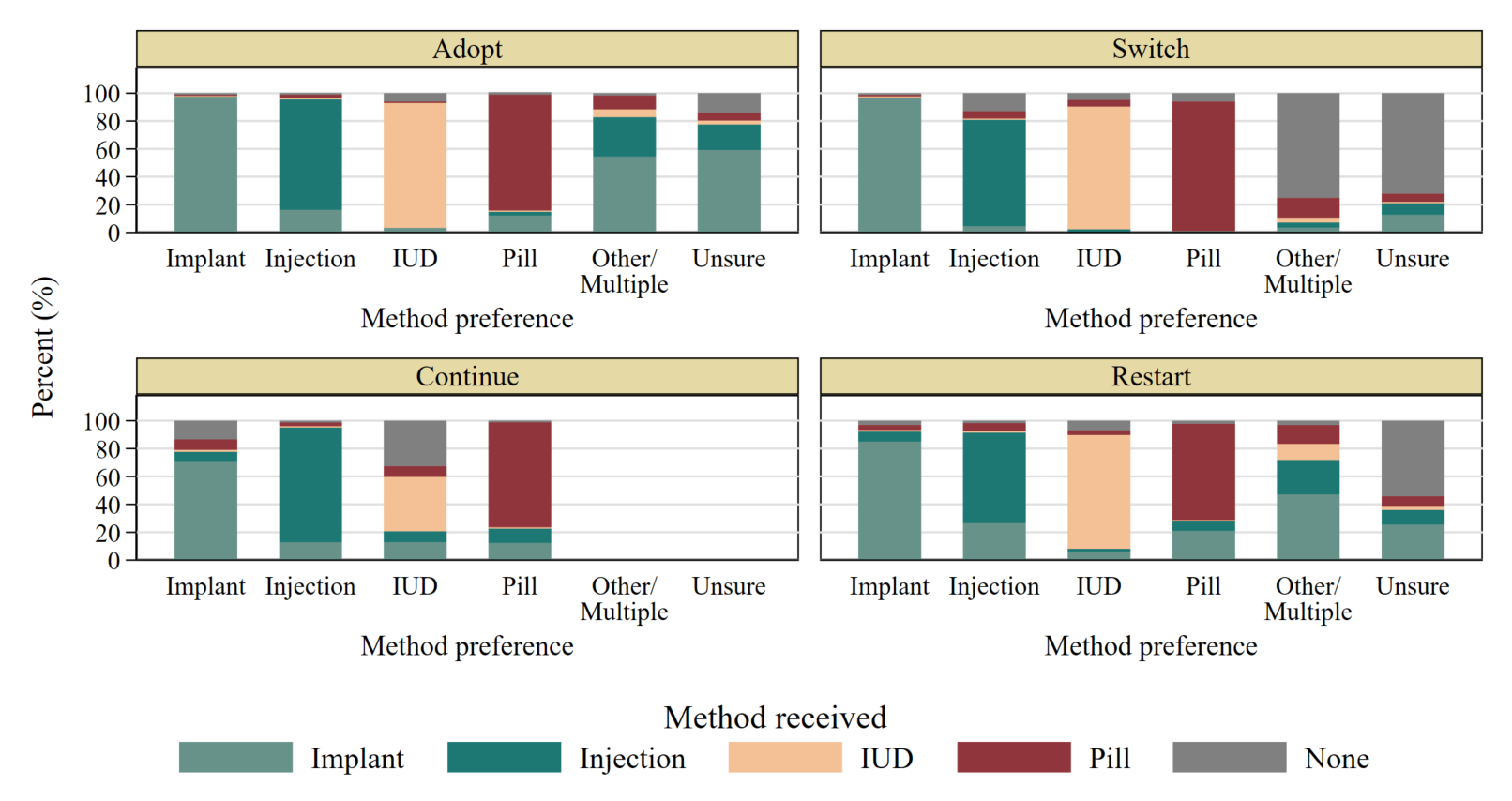


**Appendix Box 1:** List of perceived treatment index components

| Did you feel the provider you saw for family planning paid attention to you during your stay in the facility? |
| --- |
| Were you allowed to have someone you wanted to stay with you during your visit? |
| Did you feel the provider you saw for family planning cares about you as a person? |
| Did you feel you could completely trust the provider you saw for family planning with regards to your care? |
| In general, did you feel safe in the health facility? |
| Did the provider answer all of your questions to your satisfaction? |
| Were you given enough information about your care in order for you to feel like you understood what happened? |
| Did you feel the provider you saw for family planning at the facility clearly explained things to you? |
| Did you feel you could ask the provider you saw for family planning at the facility any questions you had? |
| Did the provider you saw for family planning support your anxieties and fears about your family planning procedure or your choice? |
| Did the provider you saw for family planning talk to you about how you were feeling? |
| Did the provider you saw for family planning allow you to give your opinion about what you needed? |
| Did you feel listened to by the provider? |
| Did the provider you saw for family planning consider your personal situation when advising you about FP methods? |
| Did you feel like the provider you saw for family planning involved you in decisions about your FP choice? |
| Did the provider you saw for family planning allow you to give your opinion about what you needed? |
| Did the provider you saw for family planning give you enough information to make the best decision about your birth control method? |
| Did the provider you saw for family planning give you the time you needed to consider the contraceptive options they discussed? |
| Did the provider you saw for family planning let you say what mattered to you about your FP method? |
| Did the provider make an effort to make sure that others in the facility could not see or hear your conversation with her/him? |
| Do you feel like your personal information was or will be kept confidential at this facility? |
| Did the provider you saw for family planning treat you with disrespect? |
| Did the provider you saw for family planning treat you in an unfriendly manner? |
| Did you feel the provider judged you? |
| Did you feel the provider scolded you? |
| Did the provider make you feel uncomfortable because of your sex life (e.g., when I started having sex, my sexual preferences, the number of partners I have, the number of children I have)? |
| Did the provider pressure you to use the method they wanted you to use? |
| When meeting with the provider during your visit, do you think other clients could see you? |
| When meeting with the provider during your visit, do you think other clients could hear what you said? |

**Appendix Box 2**: List of perceived person-centeredness components

| **Item** |
| --- |
| Did the provider you saw for family planning allow you to give your opinion about what you needed? |
| Did the provider you saw for family planning consider your personal situation when advising you about FP methods? |
| Did the provider you saw for family planning give you the time you needed to consider the contraceptive options they discussed? |
| Did you feel the provider judged you? |
| Did you feel the provider scolded you? |
| Did the provider make you feel uncomfortable because of your sex life? |
| When meeting with the provider during your visit, do you think other clients could see you? |
| Did the provider pressure you to use the method they wanted you to use? |
| Did you feel the provider you saw for family planning cares about you as a person? |
| Did you feel like the provider you saw for family planning involved you in decisions about your FP choice? |
| Was the provider interested in your opinions? |
